# Comparison and Analysis of Diffusion Models: Growth Kinetics of Diiron Boride Layers on ASTM A283 Steel

**DOI:** 10.3390/ma15238420

**Published:** 2022-11-26

**Authors:** Martín Ortiz-Domínguez, Oscar Armando Gómez-Vargas, Mariana Bárcenas-Castañeda, Víctor Augusto Castellanos-Escamilla

**Affiliations:** 1 Ingeniería Mecánica, Escuela Superior de Ciudad Sahagún, Universidad Autónoma del Estado de Hidalgo, Carretera Cd. Sahagún-Otumba s/n, Zona Industrial, Ciudad Sahagún 43990, Hidalgo, Mexico; 2 División de Estudios de Posgrado e Investigación, Instituto Tecnológico de Tlalnepantla, TecNM, Av. Instituto Tecnológico, S/N. Col. La Comunidad, Tlalnepantla de Baz 54070, Estado de Mexico, Mexico; 3 División de Ingeniería Química y Bioquímica, Tecnológico de Estudios Superiores de Ecatepec, TecNM, Av. Tecnológico S/N, Col. Valle de Anáhuac, Ecatepec de Morelos 55210, Estado de Mexico, Mexico

**Keywords:** boronizing, layer growth, diffusion models, Fe_2_B, minimum energy

## Abstract

Hard-coated surfacing of a few micrometers is widely applied to increase the efficiency of tools, e.g., for cutting, forming, and casting applications. Therefore, the base thermodiffusion surface treatment is a practical solution to these issues by hardening surface layers with interstitial elements such as carbon, nitrogen, and boron. In particular, within this study, the growth kinetics of an iron boride layer on ASTM 283 steel were investigated with two diffusion models of the powder-pack boriding technique in the temperature range of 1123–1273 K with different treatment periods. The first model, called the steady-state diffusion model, used the modified version of the mass balance equations at the Fe_2_B/substrate growth interface, the parabolic growth law, and the solution of Fick’s second law without time dependence. At the same time, the second diffusion model was based on Goodman’s method, also called the integral heat balance method. Afterward, the diffusion coefficient of boron in the Fe_2_B phase was calculated by fitting the experimental data to the models. Nevertheless, the estimated value for the activation energy of ASTM A238 steel in both diffusion models was coincident (168.2 kJ∙mol^−1^). A mathematical analysis was implemented by means of a power series (Taylor series) to explain this similarity. The SEM examinations showed a solid tendency to saw-tooth morphology at the growth interface with the formation of the Fe_2_B layer, whose presence was verified by XRD analysis. The tribological characterizations, including the tests of Rockwell-C indentation, pin-on-disc, and Vickers hardness test method, were used to analyze the antiwear features of the Fe_2_B layers. Finally, this value of energy was compared to the literature for its experimental validation.

## 1. Introduction

The layers formed by thermal diffusion in surface engineering show high adhesion due to the strong bonds generated between the coating phases and the base material [1,2,3]. Particularly, in the boriding process, the diffusion of boron atoms into the substrate can occur through two main diffusion mechanisms: (i) the interstitial diffusion mechanism occurs because the atomic radius of boron (0.87 × 10^−12^ m) is considerably smaller than that of iron (155.8 × 10^−12^ m), and (ii) diffusion through vacancies is generated because the substrate has structural defects on the surface, which develop especially at elevated temperature. In this framework, the boriding treatment, which is one of the surface hardening processes, is related to the thermodiffusion of boron atoms on the steel surface to produce the boronized layers [4]. The boriding process is performed in the range of 800–1050 °C for 0.5–10 h in which the active boron can be delivered from various supplies with diverse physical states: solid [5,6], liquid [7], gas [8], plasma [9], and plasma-paste [10]. Powder-pack boriding provides benefits such as ease of processing, low investment in equipment, and cost–effectiveness [11]. For iron-based alloys, two forms of iron borides (FeB and Fe_2_B) can be generated under equilibrium conditions. The Fe_2_B layer is more convenient for industrial use than the dual-phase layer (FeB + Fe_2_B) due to the risk of cracks spreading along the interface (FeB/Fe_2_B). In the industry, ASTM A283 Grade C is used for general purpose structural applications of medium strength requirement. Its machinability is good, as contrasted with low carbon steels. Many diffusion models related to the kinetics of diiron boride layers deposited on different substrates have been published in the literature [5,12,13,14,15,16,17,18,19,20]. The application of these simulation tools for operational purpose is aimed at optimizing the thickness of the Fe_2_B or FeB + Fe_2_B layer and prolonging the service life of the processed steels. The points of convergence and differences noted in these models are reviewed in what follows. Campos et al. [5] employed the mass conservation at the (Fe_2_B/substrate) by underestimating the boride incubation in the example of the paste boronizing agent of pure iron. This model assumed the ratio between the molar volume of the Fe_2_B phase and that of the iron phase. The boride incubation times were considered by introducing a dependent temperature parameter in this model in which the boron concentration profile is parabolic. Türkmen et al. [6] implemented the Goodman-method-based approach for the dynamic of Fe_2_B layers in SAE steel employing boric acid as the boron source. This selected method was experimentally confirmed for a supplementary boronizing condition (6 h at 1198 K). Morgado-González et al. [13] implemented the mean diffusion coefficient model for the growth of the diiron boride layer on ASTM A1011 steel with the existence of boride incubation periods. In this model, the distribution of boron content within the Fe_2_B phase was linear. The fuzzy logic technique with two methods (fuzzy logic Mamdani and Takagi-Sugeno) was adopted by Campos et al. [14] as another tool for predicting the paste-boriding process of AISI 1045 steel. In this case, Fe_2_B layers were formed at the surface of this steel by varying the thickness of the boron paste from 1 to 5 mm. When plotting the experimental thicknesses against the predicted values, the average errors determined were, respectively, 2.61% and 2.61% for the Mamdani and Takagi-Sugeno methods.

López-Perrusquia et al. [15] conducted the simulation work on the pack-boriding of spheroidal graphite in cast iron ASTM A-536 grade 80-56-06. They generated the diiron boride layers within the temperature range of 1173 to 1273 K with 6, 7, 8, and 10 h. The mass conservation at the moving interface was considered with the occurrence of incubation times. It was experimentally verified for two boriding temperatures (1123 and 1273 K with an exposure time of 10 h) and an acceptable agreement was obtained between the experimental layers and the prediction results. Ortiz-Dominguez et al. [16] used two different diffusion approaches for simulating the boriding dynamic of Fe_2_B layers on AISI 1018 steel (with the classical model for diffusion and the dimensional analysis). These two models were finally confirmed for four additional conditions of the process (5 h each temperature of the process). Abdellah et al. [17] proposed an approach which is a comprehensive version of the model proposed by Campos-Silva [19] and is related to the evolution of boride layers on AISI 1018 steel. This diffusion model referred to the alternative diffusion model. It was employed to analyze the growth of diiron boride layer on ASTM A36 steel by introducing a dimensionless parameter accounting for the boride incubation times. In this model, the migration of the boron atoms in the iron phase was considered with the consideration of incubation periods of Fe_2_B phase. From another angle, Ramdan et al. [18] employed the multiphase field modeling to investigate the textured growth of Fe_2_B needles on the steel substrate. The simulation results confirmed, experimentally, the preferred orientation of boride needles in the normal direction of the material’s surface which was independent of their initial size. Milinovi’c et al. [20] suggested a new diffusion model to the additional description according to the analysis of the volume fraction of the boride phase in the cross-section percentage by volume of the boride layer in the cross-section to estimate the average boride coating thickness. This mathematical diffusion model offers several advantages over classical or conventional methods: the thickness of the boride layer can be calculated rooted on the depth, and it is possible to separate the compacted and the saw-tooth layer, which allows the impact of the parameters on the morphology of the boride layer to be controlled. In this study, original data about the kinetics of diiron boride (Fe_2_B) layers on ASTM A283 steel were used for simulation purposes by adopting the mass conservation equation at the Fe_2_B/Fe growth interface without time dependence and integral method [12,17]. Till now, no simulation study was conceived on this type of borided steel. The formed Fe_2_B layers on ASTM A283 steel were characterized by different experimental techniques to investigate their tribological performance for the given boriding conditions. In addition, the adopted model was exploited to assess the diffusivities of boron in Fe_2_B in the interval of 1123 to 1273 K. The boron minimum energy for ASTM A283 steel was determined through the plot of Arrhenius law and compared with the results of the literature. Finally, the same models were verified experimentally for two additional boriding conditions (1223 and 1273 K for a time duration of 9 h).

## 2. Materials and Methods

### 2.1. Materials and Boriding Treatment

**Figure 1:**
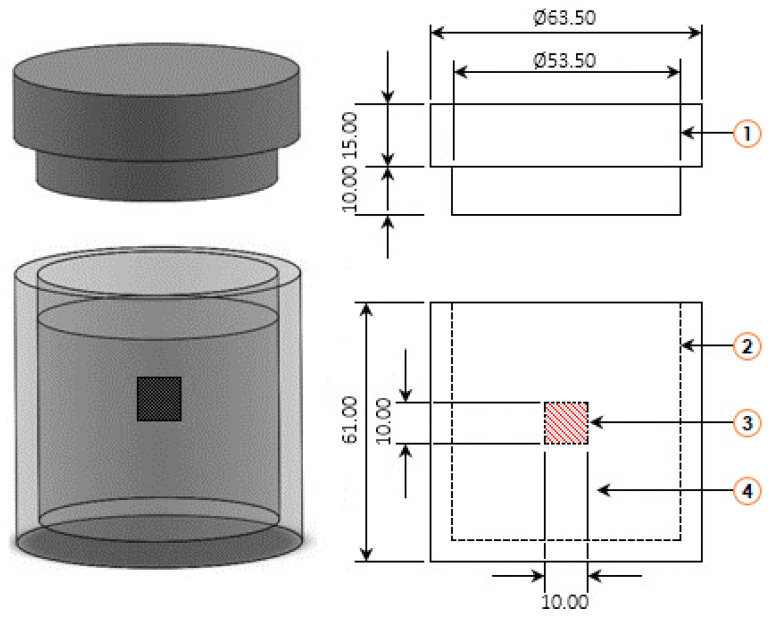
Schematic and cross-sectional representation of the AISI 316L stainless steel container used for the thermochemical diffusion process. (1: lid with a relief hole; 2: powder mixture (B_4_C + KBF_4_ + SiC); 3: substrate; 4: steel container) (the scale is in millimeters).

The ASTM A283 steel to be treated had chemical elements of 0.24% C, 0.09% Mn, 0.035% P, 0.04% S, 0.20% Cu, and 0.15–0.40% Si. The samples to be boronized had a cubic configuration with 0.01 m length on its sides. Before this thermochemical process, the samples were subjected to grinding by using wet-dry silicon carbide (SiC) sandpaper of 80 to 2500 grit and immersion in an ultrasonic bath filled with a binary liquid solution composed of mixing n-heptane and ethanol for 1200 s. The substrates were placed inside a stainless steel container and embedded in the powder mixture (see Figure 1) whose chemical composition expressed in weight percent is the following: (33.5% B_4_C, 5.4% KBF_4_, and 61.1% SiC).

Such a chemical composition enables us to generate a single boride layer (Fe_2_B) at the surface of the treated surface [13]. A conventional furnace of brand Nabertherm N 250/85 HA was employed for heating the treated samples at the considered temperatures for different time durations by using argon gas as a protective atmosphere. The temperature range selected was 1123 to 1273 K with exposure times of 2, 4, 6, and 8 h. When the selected temperature was reached, the borided specimens were removed from the electric furnace and were cooled in up to room temperature.

### 2.2. Description Tools

**Figure 2:**
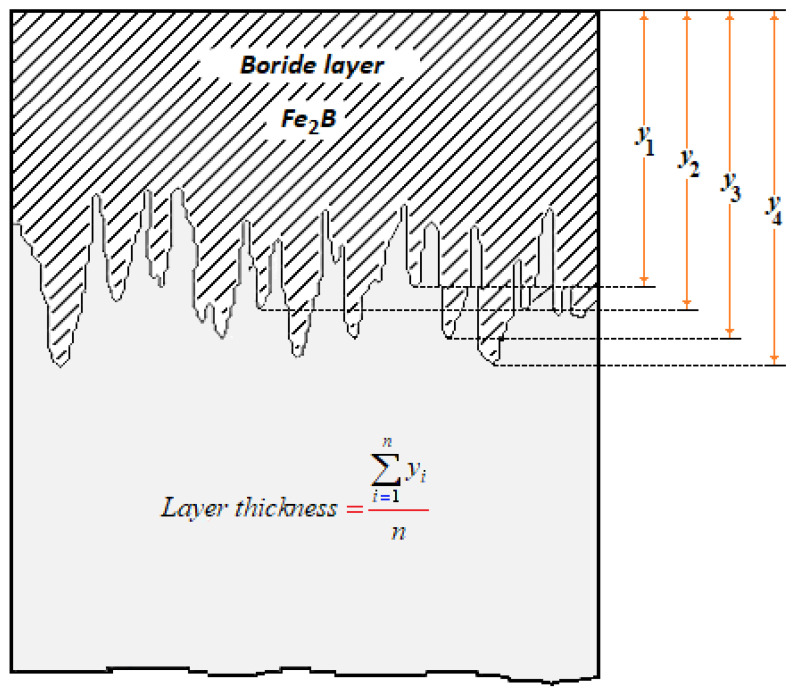
Method of measurement of layer thicknesses of Fe_2_B.

The representative sections of the borided samples were cleaned and polished up to a mirror finish according to the ASTM metallography standard procedure for SEM examinations with Quanta 3D FEG-FEI JSM7800-JOEL (Akishima, Tokio, Japan). A Nital solution of 4 vol.% was used to etch the observed surfaces. An automatic procedure for measuring the thicknesses of diiron boride layers was performed using an MSQ PLUS software. Sixteen borided specimens were considered, with one replicate for each of them. Likewise, fifty measurements were made in each of the thirty-two cross-sections selected to determine average layer thickness values (see Figure 2).

The microhardness profile across the boronized layer was determined using a Vickers hardness tester with a pyramidal diamond indenter selecting a load of 50 and a dwell time of 15 s. With the help of the Rockwell-C indentation test, the adhesion quality of the boride layer with respect to the substrate was determined, complying with the VDI 3198 norm [21].

In this test, 1471 N was applied as the test load to assure the impairment of the boride coatings around the area of the indentation [22]. Only three indentations were made for each of the borided sample to assess the adhesion nature of the boride coatings. The generated craters under the indentation zones were viewed using a scanning electron microscope. For the tribological behavior of the boride coatings and substrate, the pin-on-disc sliding wear test was performed using a CSM tribometer in unlubricated condition [22]. Tribological tests were performed considering the following parameters: a linear velocity of 8 × 10^−2^ m/s, a sliding distance of 500 × 10^3^ mm, a test load (P = 5 N), a radial distance (r = 14 mm), and a relative humidity of 40 vol.% at ambient temperature [22]. For estimating the average value of the wear depth in each groove generated on the surface of the wear track, we used a Mitutoyo Surftest SJ-301 surface roughness tester with a diamond stylus tip.

### 2.3. First Approach: Steady-State Diffusion Model

**Figure 3:**
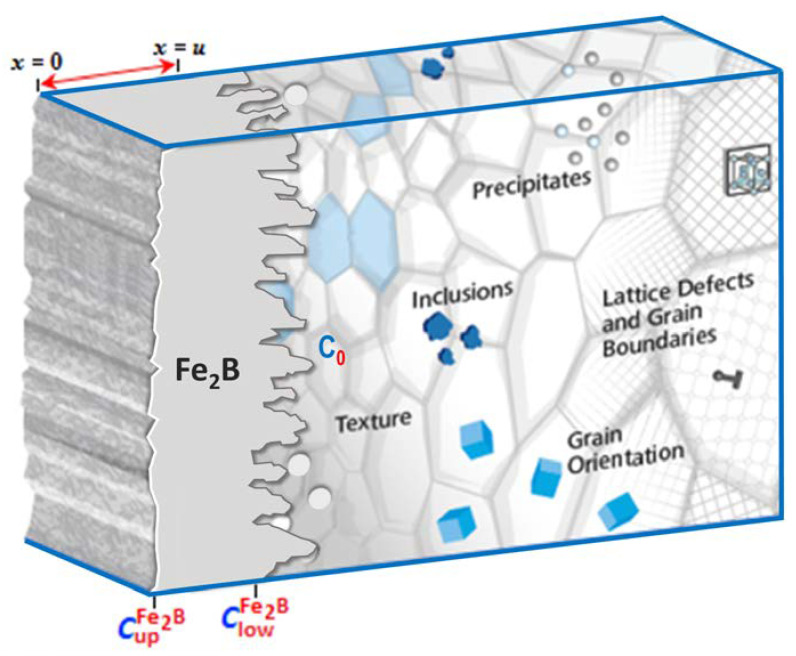
Schematic representation of the Fe_2_B layer created on the surface of ASTM A283 steel.

The steady-state diffusion model [23] was used for studying the growth kinetics of the diiron boride layer on ASTM A283 steel (see Figure 3).

**Figure 4:**
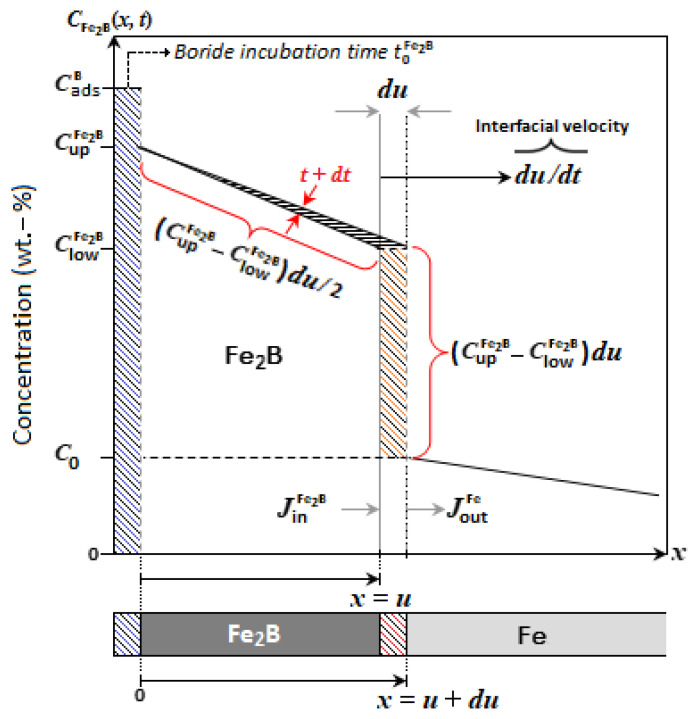
Schematic boron concentration profile through the Fe_2_B layer.

**Figure 5:**
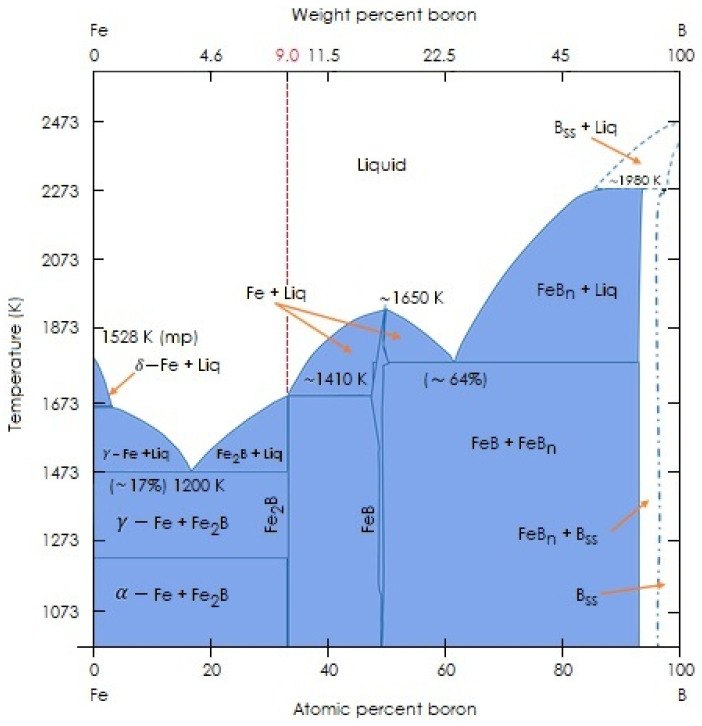
Phase diagrams of the Fe-B system.

A schematic diagram of the boron concentration profile along the Fe_2_B layer is shown in Figure 4. u refers to the layer thickness of the Fe_2_B phase, $C_{up}^{Fe_{2}B}$ indicates the upper boron concentration in Fe_2_B (=9 wt.% B) by the phase diagram of the Fe-B system of Figure 5, and $C_{low}^{Fe_{2}B}$ corresponds to the lower boron concentration in Fe_2_B (=8.83 wt.% B). The concentration values considered for both limits are because the Fe_2_B phase exhibits a very narrow concentration range (of about 1 at.% B), as reported by Brakman et al. [24]. The term $C_{0}$ (=35 × 10^−4^ wt.% B) represents the solubility limit of boron within the substrate, which can be neglected [25,26]. $C_{ads}^{B}$ establishes the adsorbed boron concentration at the substrate surface [27].

The following assumptions were made to construct the steady-state diffusion model:

(i) Once the threshold value of the boron concentration ($C_{up}^{Fe_{2}B}$) at the surface is reached, the formation of layers in flat fronts begins;

(ii) An initial Fe_2_B layer ($u_{0}$) is formed after a given incubation period ($t_{0}^{Fe_{2}B}$);

(iii) The boride layer grows as a consequence of the perpendicular diffusion of boron atoms on the substrate surface;
(iv) Fe_2_B layer formation occurs under thermodynamic equilibrium conditions;
(v) The growth kinetics are regulated by the diffusivity of boron atoms in the composition of the boride layer;
(vi) The flow of boron atoms is one-dimensional;
(vii) The boron concentration at the surface and growth interface remains constant in the Fe_2_B layer during the process;
(viii) The Fe_2_B layer is narrow in comparison with the thickness of the sample;
(ix) The temperature at each point of the sample is identical during the whole process;
(x) The chemical potential does not vary with time.

According to the above assumptions, and considering Figure 4, the initial and boundary conditions can be expressed as:

Initial state:

(1) $$In\;t=0,\;x>0,with:C_{Fe_{2}B}\left(x\right)=C_{0}=35\times 10^{-4}\;wt.\%.$$

Boundary conditions:

(2) $$C_{Fe_{2}B}\left(x=u_{0}\approx 0\right)=C_{up}^{Fe_{2}B}=9\;wt.\%,\;\mathrm{for}\;C_{ads}^{B}>9\;wt.\%,$$

(3) $$C_{Fe_{2}B}\left(x=u\right)=C_{low}^{Fe_{2}B}=8.83\;wt.\%,\;\mathrm{for}\;C_{ads}^{B}<8.83\;wt.\%.$$

The mass balance equation at the growth interface (Fe_2_B/substrate) is defined by the inward flow (${\vec{J}}_{Fe_{2}B}{\left[x(t)\right]}_{x\;=\;u}\hat{i}=-D_{Fe_{2}B}{\left\{dC_{Fe_{2}B}\left[x(t)\right]/dx\right\}}_{x\;=\;u}\hat{i}$) and the outward flow (${\vec{J}}_{Fe}{\left[x(t+dt)\right]}_{x\;=\;u+du}\hat{i}=-D_{Fe}{\left\{dC_{Fe}\left[x(t+dt)\right]/dx\right\}}_{x\;=\;u+du}\hat{i}=0$). Thus, this equation can be written as:

(4) $$\left(\frac{C_{up}^{Fe_{2}B}+C_{low}^{Fe_{2}B}-2C_{0}}{2}\right){\left(\frac{dx}{dt}\right)|}_{x\;=\;u}=-D_{Fe_{2}B}{\left(\frac{dC_{Fe_{2}B}\left(x\right)}{dx}\right)|}_{x\;=\;u}.$$

Fick’s second law determines the distribution of boron atoms through the boride layer without consideration of the time ($\nabla^{2}C_{Fe_{2}B}(x)=0$) and can be formulated as follows

(5) $$C_{Fe_{2}B}\left(x\right)=\frac{C_{low}^{Fe_{2}B}-C_{up}^{Fe_{2}B}}{u}x+C_{up}^{Fe_{2}B}.$$

The Fe_2_B thickness is represented by Equation (6):

(6) $$u\left(t\right)=2\varepsilon D_{Fe_{2}B}^{1/2}{\left(t-t_{0}^{Fe_{2}B}\right)}^{1/2},$$

where ε is a constant without unit, $t_{0}^{Fe_{2}B}$ is the boride incubation time, and t the exposure time. Using the above Equations (5) and (6) in Equation (4), we obtain the following:

(7) $$\varepsilon^{2}=\frac{C_{up}^{Fe_{2}B}-C_{low}^{Fe_{2}B}}{C_{up}^{Fe_{2}B}+C_{low}^{Fe_{2}B}-2C_{0}}=9.5\times 10^{-3}.$$

### 2.4. Second Approach: The Integral Diffusion Model

The boron concentration profile for the Fe_2_B phase with time dependence is determined by the second Fick’s law:

(8) $$\frac{\partial C_{Fe_{2}B}\left(x,t\right)}{\partial t}=D_{Fe_{2}B}\frac{\partial^{2}C_{Fe_{2}B}\left(x,t\right)}{\partial x^{2}}.$$

Goodman’s integral method [12,17] was used for studying the growth kinetics of diiron boride layer by a preliminarily defined parabolic profile $C_{Fe_{2}B}\left(x,t\right)$: namely,

(9) $$C_{Fe_{2}B}\left(x,t\right)=b\left(t\right){\left(u-x\right)}^{2}+a\left(t\right)\left(u-x\right)+c.$$

The concentration profile of Equation (9) is defined in the domain of interest, from *x* = 0 to *x* = *u*. The constant c in Equation (9) is determined by substituting Equation (3); we obtain

(10) $$C_{Fe_{2}B}\left(x=u\right)=C_{low}^{Fe_{2}B}=b\left(t\right){\left(u-u\right)}^{2}+a\left(t\right)\left(u-u\right)+c,$$

(11) $$c=C_{low}^{Fe_{2}B}.$$

Applying the boundary conditions given by Equations (2) and (3) yields

(12) $$b\left(t\right)=\frac{C_{up}^{Fe_{2}B}-C_{low}^{Fe_{2}B}-a\left(t\right)u}{u^{2}}.$$

It should be noted that the parameters in Equation (9) (a(t) and b(t)) must be strictly positive due to the negative slope of the boron concentration profile. In addition, integrating Equation (8) in the interval 0 ≤ *x* ≤ *u*, has a priori the form:

(13) $$D_{Fe_{2}B}\int_{x\;=\;0}^{x\;=\;u}\frac{d}{dx}\left(\frac{\partial C_{Fe_{2}B}(x,t)}{\partial x}\right)dx=\int_{x\;=\;0}^{x\;=\;\;u}\left(\frac{\partial C_{Fe_{2}B}(x,t)}{\partial t}\right)\;dx.$$

Therefore, after some algebraic manipulations, the solution of Equation (13) is as follows:

(14) $$2D_{Fe_{2}B}b(t)u(t)=\frac{u^{2}}{2}\frac{da(t)}{dt}+a(t)u\frac{du}{dt}+\frac{1}{3}u^{3}\frac{db(t)}{dt}+b(t)u^{2}\frac{du}{dt}.$$

From Equation (14), we can determinate the diffusion coefficient; for that, it is necessary to know the parameters a(t) and b(t). Rewriting Equation (4) with time dependence, we obtain

(15) $$\left(\frac{C_{low}^{Fe_{2}B}-2C_{0}+C_{up}^{Fe_{2}B}}{2}\right){\frac{dx}{dt}|}_{x\;=\;u}=-D_{Fe_{2}B}{\frac{\partial C_{Fe_{2}B}(x,t)}{\partial x}|}_{x\;\;=\;u}.$$

Involving the boron concentration profile given in Equation (9) in the left-hand side of Equation (15), we see that

(16) $$\left(\frac{C_{low}^{Fe_{2}B}-2C_{0}+C_{up}^{Fe_{2}B}}{2}\right)\left(\frac{{\frac{\partial C_{Fe_{2}B}(x,t)}{\partial t}|}_{x\;\;=\;u}}{{\frac{\partial C_{Fe_{2}B}(x,t)}{\partial x}|}_{x\;\;=\;u}}\right)=-D_{Fe_{2}B}{\frac{\partial C_{Fe_{2}B}(x,t)}{\partial x}|}_{x\;\;=\;u}.$$

If we combine Equation (8) with Equation (16), we obtain

(17) $$\left(\frac{C_{low}^{Fe_{2}B}-2C_{0}+C_{up}^{Fe_{2}B}}{2}\right)\left(\frac{{D_{Fe_{2}B}\frac{\partial }{\partial x}\left(\frac{\partial C_{Fe_{2}B}(x,t)}{\partial x}\right)|}_{x\;=\;u}}{{\frac{\partial C_{Fe_{2}B}(x,t)}{\partial x}|}_{x\;\;=\;u}}\right)=-D_{Fe_{2}B}{\frac{\partial C_{Fe_{2}B}(x,t)}{\partial x}|}_{x\;=\;u}.$$

Now, replacing Equation (9) in Equation (17), we have

(18) $$a^{2}(t)=b(t)\left(C_{low}^{Fe_{2}B}-2C_{0}+C_{up}^{Fe_{2}B}\right).$$

Similarly, substituting Equation (9) into Equation (15), we have

(19) $$\left(\frac{C_{low}^{Fe_{2}B}-2C_{0}+C_{up}^{Fe_{2}B}}{2}\right){\frac{dx}{dt}|}_{x\;\;=\;v}=D_{Fe_{2}B}a(t).$$

Combining Equation (12) with Equation (18), we obtain

(20) $$a(t)=\frac{\left(C_{low}^{Fe_{2}B}-2C_{0}+C_{up}^{Fe_{2}B}\right)\left(\sqrt{1+4\left(\frac{C_{up}^{Fe_{2}B}-C_{low}^{Fe_{2}B}}{C_{up}^{Fe_{2}B}+C_{low}^{Fe_{2}B}-2C_{0}}\right)}-1\right)}{2u}.$$

Using the above Equations (6) and (20) in Equation (19), we obtain the following:

(21) $$\varepsilon^{2}=\frac{\left(\sqrt{1+4\left(\frac{C_{up}^{Fe_{2}B}-C_{low}^{Fe_{2}B}}{C_{up}^{Fe_{2}B}+C_{low}^{Fe_{2}B}-2C_{0}}\right)}-1\right)}{2}\approx 9.4\times 10^{-3}.$$

## 3. Results

### 3.1. SEM Examinations of the Cross-Sectional Views of Borided Coatings

**Figure 6:**
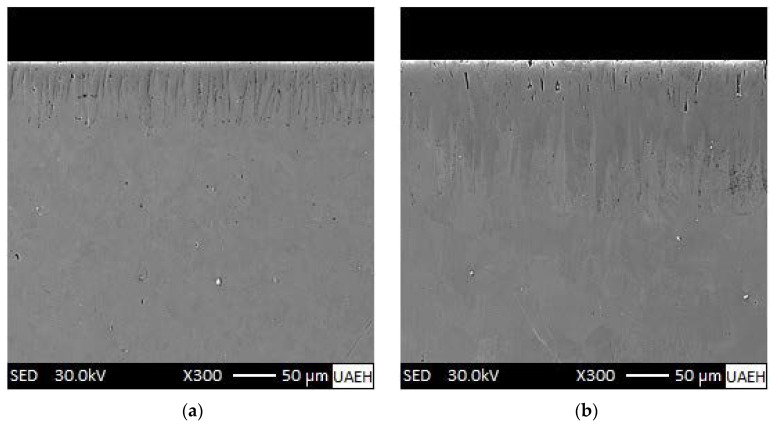

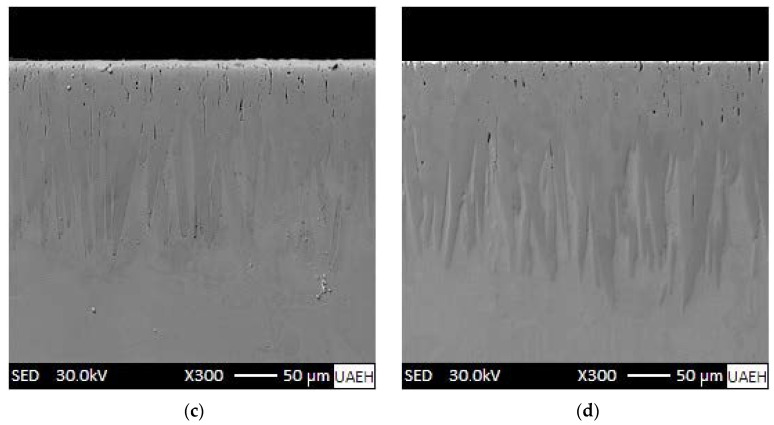
Scanning electron microscopy (SEM) images of the borided samples from ASTM A283 under the following conditions: (**a**) 1223 K for 2 h, (**b**) 1223 K for 4 h, (**c**) 1273 K for 6, and (**d**) 1273 K for 8 h.

**Figure 7:**
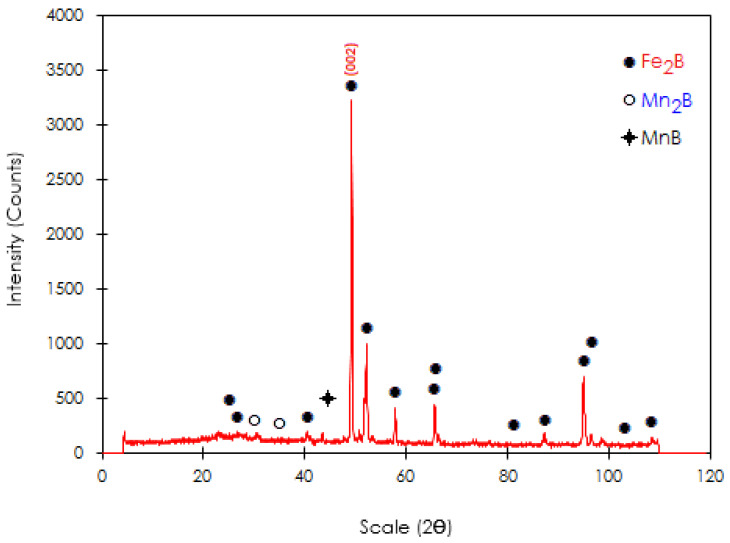
XRD peak profile produced at the surface of the borided ASTM A283 steel. The selected temperature of 1273 K for 8 h of exposure time.

Figure 6 shows SEM observations, which revealed that many borided needles are of dendritic nature, as reported by Ninham and Hutchings [28]. The columnarity of this interface is the result of side-arm growth, similar to that observed during the solidification of ferrous and nonferrous alloys [29]. Concerning the nature of boride coatings, Palombarini and Carbucicchio [30] informed that the sawtooth morphology of the interface (Fe_2_B/substrate) in low-alloy steels could be explained by enhanced growth at the tips of boride needles. Likewise, Martini et al. [31] studied the growth mechanism of iron borides. The results of peak profile analysis in X-ray diffraction and metallographic observations made them conclude that the Fe_2_B phase is the first product to form with a growth mechanism involving three stages. In the first stage, the first acicular crystals of Fe_2_B form a surface layer of randomly oriented crystals. In the second stage, as the metal surface is coated, more Fe_2_B crystals come into contact with adjacent crystals and are forced to grow inward into the metal, retaining an acicular shape. Finally, in the third growth stage all Fe_2_B boride needles tend to grow perpendicular to the external surface, establishing a strong texture of the Fe_2_B phase in the crystallographic orientation (002), which is congruent with the result presented in Figure 7 of the XRD pattern for a borided sample of ASTM A283 steel for 8 h at 1273 K, where the largest diffraction peak corresponds precisely to the crystallographic orientation (002) of the Fe_2_B phase. Such morphology of the interface between the boride layer and the substrate is shown in pure iron [5,32] and either low- or medium-carbon alloys [6,12,13,16]. Another model to explain the mechanism of texture formation in iron boride layers on low-carbon steels was proposed by Zhong et al. [33] who applied powder-pack boriding treatment to low-carbon steel using only a temperature of 950 °C and a time of 6 h; the microstructure of the obtained phase was characterized by electron backscattered diffraction (EBSD). The EBSD images confirmed the presence of the Fe_2_B phase (with a tetragonal structure), with sporadic contributions from the FeB phase around the surface. The Fe_2_B phase is perceived to have a sawtooth morphology. Furthermore, the authors stated that the Fe_2_B grain growth (with the following lattice parameters: a = 0.5088 nm, b = 0.5088 nm, and c = 0.4232 nm) does not take place around the grain boundaries of the substrate as previously believed but penetrates through the substrate crystals. In the inverse pole figure map in the Z direction, it can be appreciated that about 80% of the grains of the two borided phases (FeB and Fe_2_B) are close to {010}, and the rest are around {010} for FeB and {110} for Fe_2_B. In addition, in the inverse pole figure in the Z direction (normal direction), it is observed that Fe_2_B shows a strong texture near {010}. Borides grow by the stacking of crystalline planes {001}. The boride phases (FeB and Fe_2_B) show columnar morphologies and a preferential direction of growth for both phases is [001].

Furthermore, the generated boride layer’s thickness ranged between 73 ± 13 µm for 2 h and 115 ± 16 µm for 4 h for the same boriding 1223 K. Likewise, 194 ± 25 µm for 6 h and 226 ± 35 µm for 8 h for the same boriding 1273 K. It is seen the layers’ thicknesses are influenced by the increase in the period of time for a fixed boriding temperature. In fact, a study by VillaVelázquez-Mendoza et al. [32] showed that the process temperature had a bigger contribution (about 67%) than the treatment time (with a percentage of about 16%) on the kinetics of boronized layers on AISI 1018 steel based on ANOVA analysis.

Nevertheless, boriding generally results in forming a needle-like microstructure of the Fe_2_B phase, as mentioned above. This microstructure makes the boride layer substantially brittle, leading to poor performance of parts hardened with this technique when subjected to high impact and local loads [34]. As an alternative to the breakdown of the needle-like microstructure on the surface, Lin and Han [35] proposed a novel combination of two surface treatments (flame-spray and boriding) that allows for avoiding the needle-like microstructure while substantially enhancing the ductility and fracture toughness on the surface. Three different boriding media were used (B1, B2, and B3) with different temperatures and times. B1 was the best option to generate layers with high hardness values and wear and corrosion resistance, which is attributed to the formation of (Fe_x_Cr_y_Ni_1−x−y_)_2_B. The formation of Me_2_B (Me = Fe, Ni, etc.) is considered the most favorable microstructure in boriding materials because Me_2_B incorporates a combination of high hardness and compressive stresses on the hardened surface. Likewise, Eroglu [34] proposed a new process for forming boride layers produced by a shielded metal arc welding (SMAW) electrode. Three electrodes with different amounts of ferroboron were considered for the treatment in the electrode shield. The micrographs obtained by scanning electron microscopy (SEM) of the coating with electrode I on sample I (with a boride coating thickness of 4.1 mm and a eutectic microstructure (Fe_2_B + α-Fe) + perlite) presented a hypoeutectic structure (an eutectic with sorbitic perlite islands). X-ray analysis results confirmed that the eutectic structure is formed by Fe_2_B and α-Fe. The microstructure of the coating is modified concerning the boron content. As the boron content in the coating increases, the microstructure changes from hypoeutectic to hypereutectic (Fe_2_B borides and a small amount of Fe_2_B-martensite eutectic). In addition, the microhardness of the produced coatings increases relative to the boron. Finally, the coatings produced by both processes (flame spray coating-boriding and shielded metal arc welding electrode) generate a growth front (Fe_2_B/substrate) that tends to be flat, unlike the powder-pack boriding process, which generates layers with a sawtooth morphology at the interface.

### 3.2. Peak Profile Analysis in X-ray Diffraction

Figure 7 shows the peak profile in X-ray diffraction from the analyzed surface of the boronized sample at 1273 K for 8 h. It reveals the characteristic peaks of the diiron boride phase manifesting a difference in diffracted intensities with the strongest peak pertaining to the crystallographic plane (002).

The intensities of the different peaks are different and confirm the presence of the Fe_2_B phase. Likewise, the XRD spectrum revealed that other phases had been identified in this analysis (Mn_2_B and MnB). The phase composition of boride layers was shown to be dependent on the amount of boron available in the powder mixture for solid boriding [5,6,12,13,36] and also on the nature of the boriding agent for other boriding processes [8,10].

### 3.3. Microhardness Vickers Profile

**Figure 8:**
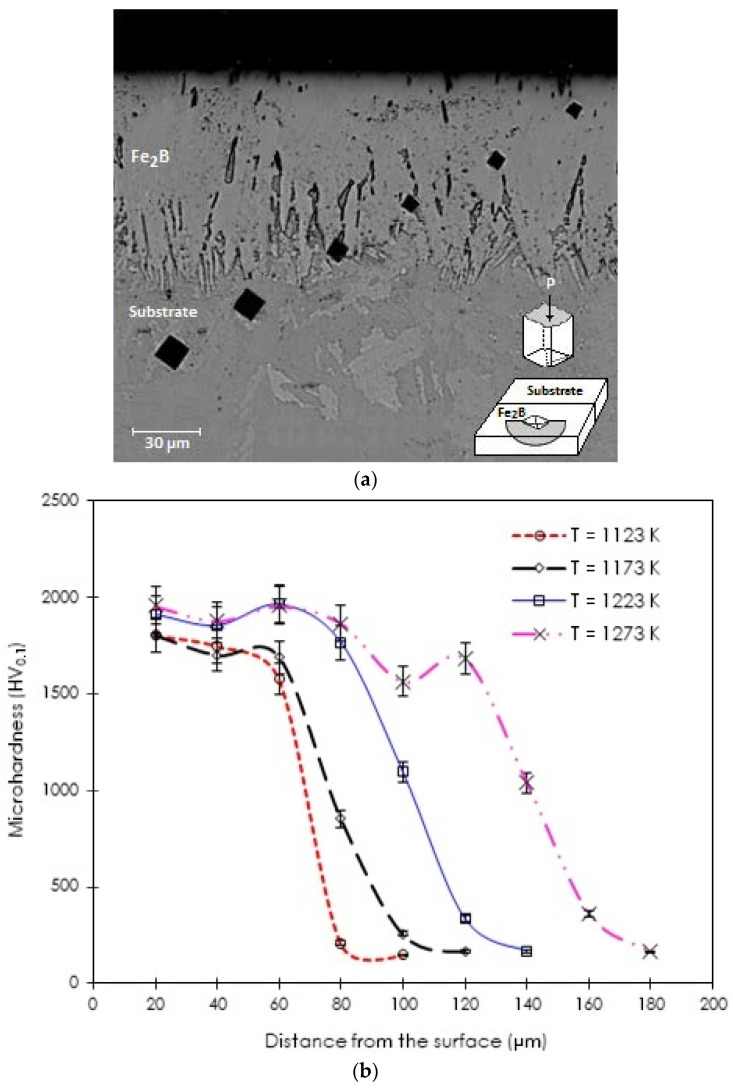
(**a**) Vickers hardness profile along the hardened layer by boriding for a borided sample of ASTM A283 steel for 4 h at 1173 K. (**b**) Describes the evolution of Vickers hardness depending on the displacement from the material surface towards its core for 8 h at 1123, 1173, 1223, and 1273 K (P = 98.1 × 10^–2^ N).

The establishment of the Vickers hardness profile along the hardened layer by boriding is an indication of a successful treatment. In Figure 8a, the indentations left by the Vickers indenter on the optical micrograph of the cross-section of the borided sample at 1173 K for 4 h are displayed. The measured surface hardness value was close to 1804 HV_0.1_, indicating the presence of a hard boride layer at the surface of ASTM A283 steel while the Vickers hardness in the material core was approximately 163 HV_0.1_. Figure 8b illustrates the evolution of Vickers hardness depending on the displacement from the material surface towards its core. The Vickers hardness values progressively decreased with the diffusion depth.

### 3.4. Rockwell-C Cohesion Indentation Tests

**Figure 9:**
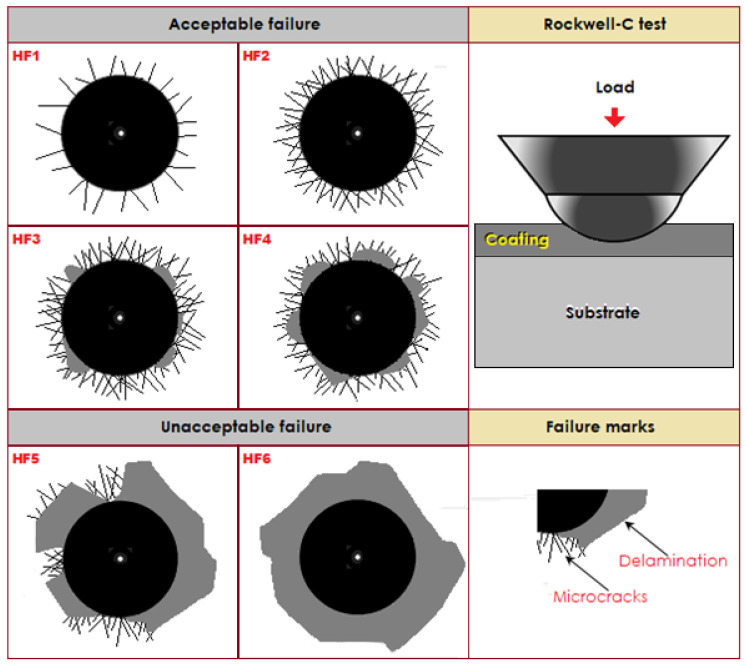
Different cohesive strength quality configurations to assess the cohesion of the boronized layer attached to the substrate by Rockwell-C indentation test.

The tribological tests are required to assess the cohesion of the boronized layer attached to the substrate. Figure 9 presents the different cohesion strength quality configurations (HF1–HF6) with which the damage of the boride layer was compared. Usually, sufficient cohesion for the boride layer is considered for the HF1–HF4 configurations, while the HF5 and HF6 are deemed insufficient. The craters obtained from the tests were compared with the 3198 standard endorsed by the Association of German Engineers’ Standards (Verein Deutscher Ingenieure Normen) [21].

**Figure 10:**
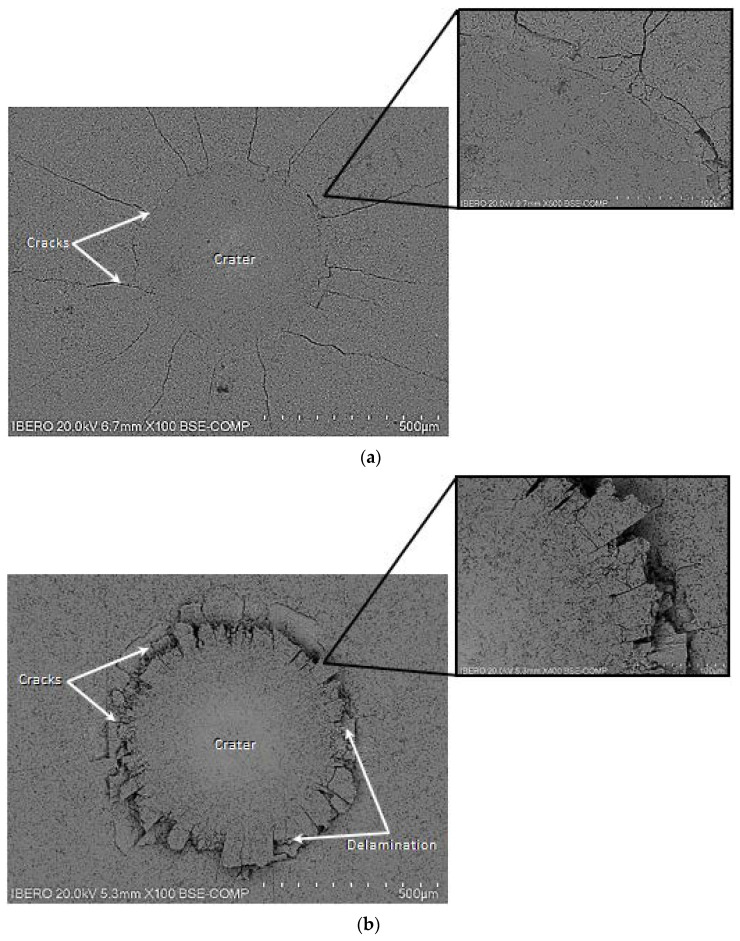
SEM micrographs of indented surfaces of treated ASTM A283 steel exhibiting the damage generated on layers formed for: (**a**) 2 h at 1123 K and (**b**) 8 h at 1273 K.

Therefore, the Daimler-Benz cohesion tests were performed on the surfaces of treated ASTM A283 steels for 8 h at 1123 and 1273 K. In Figure 10, the scanning electron microscopy (SEM) images of the two craters left by the applied indenter on the surfaces of treated samples are displayed.

For Figure 10a, no delamination was observed on the crater’s surface of the boronized sample at 1123 K during 2 h. It revealed only the presence of radial cracks emerging from the crater’s periphery and satisfied the HF2 category. In Figure 10b, some limited and spalled zones were revealed at the crater’s periphery with radial cracks produced on the hardened surface of the sample after 8 h at 1273 K. The cohesive quality of the Fe_2_B layer to the substrate obtained at 1273 K for 8 h complies with the acceptable HF4 category. From the literature results [21], the interfacial cohesion of the boronized layers is closely related to the phase composition in terms of ratio between the layer thickness of FeB to that of Fe_2_B and depends also on the boriding temperature. This boriding condition resulted in the increase of the FeB layer’s thickness accompanied by brittleness. In our present work, similar results were also found by Kayali and Kara [37], the Fe_2_B layers on Hardox-450 steel were tested by using the Daimler-Benz cohesion test. The result showed the best interfacial cohesion of boride layers formed at 1123 K for 2 h corresponding to the HF1 category compared with those produced at 1223 K for 6 h and following the unacceptable HF5 category.

### 3.5. Pin-on-Disc Tests

**Figure 11:**
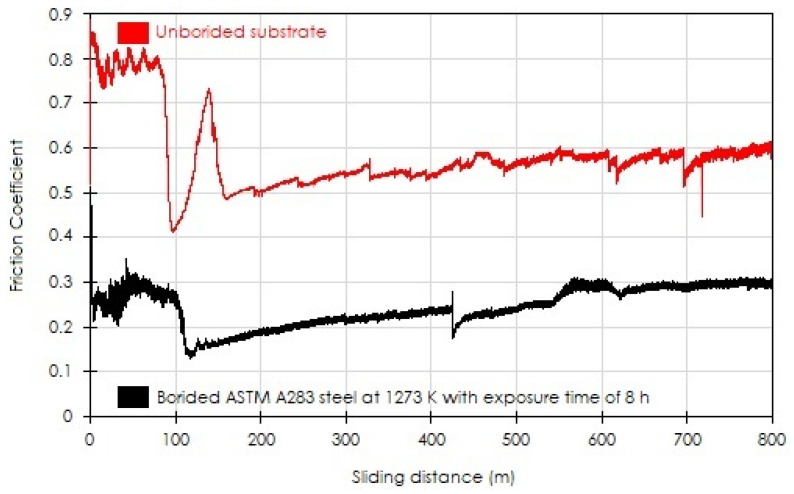
The coefficient of friction (COF) depends on the sliding length on the surfaces of untreated and boronized ASTM A283 steel for 8 h at 1273 K.

The pin-on-disc sliding wear test was employed to evaluate the coefficients of friction while sliding on the tested surfaces of untreated and treated samples. The friction coefficient stands for the ratio of normal force to tangential force and can be studied by following its variation with the sliding distance. Figure 11 describes the change in the friction coefficient with the sliding distance for the borided sample at 1273 K for 8 h and the untreated substrate of ASTM A283 steel. It can be seen that the formation of a hard coating on the surface of ASTM A283 decreased the value of the friction coefficient compared with the untreated state. The average value of the friction coefficient was in the range of 0.8–0.6 for the unborided sample and between 0.27 and 0.29 for the boronized sample. The obtained results were, in most cases, concordant with other published data in the literature [13,24,36,38], whatever the boriding parameters.

However, Türkmen and Yalamaç [6] reported contradictory results by employing different compositions of powder mixtures on pack-borided SAE 1020 steels at 950 °C for 4 h. The obtained values of friction coefficients ranged from 0.6 to 0.7 for the borided samples (at 850 °C for 4 h) and 0.6 for the unborided sample. Such a situation was attributed to the effect of porosity, surface roughness, and the presence of oxides (acting as a solid lubricant) on the evolution of the friction coefficient.

**Figure 12:**
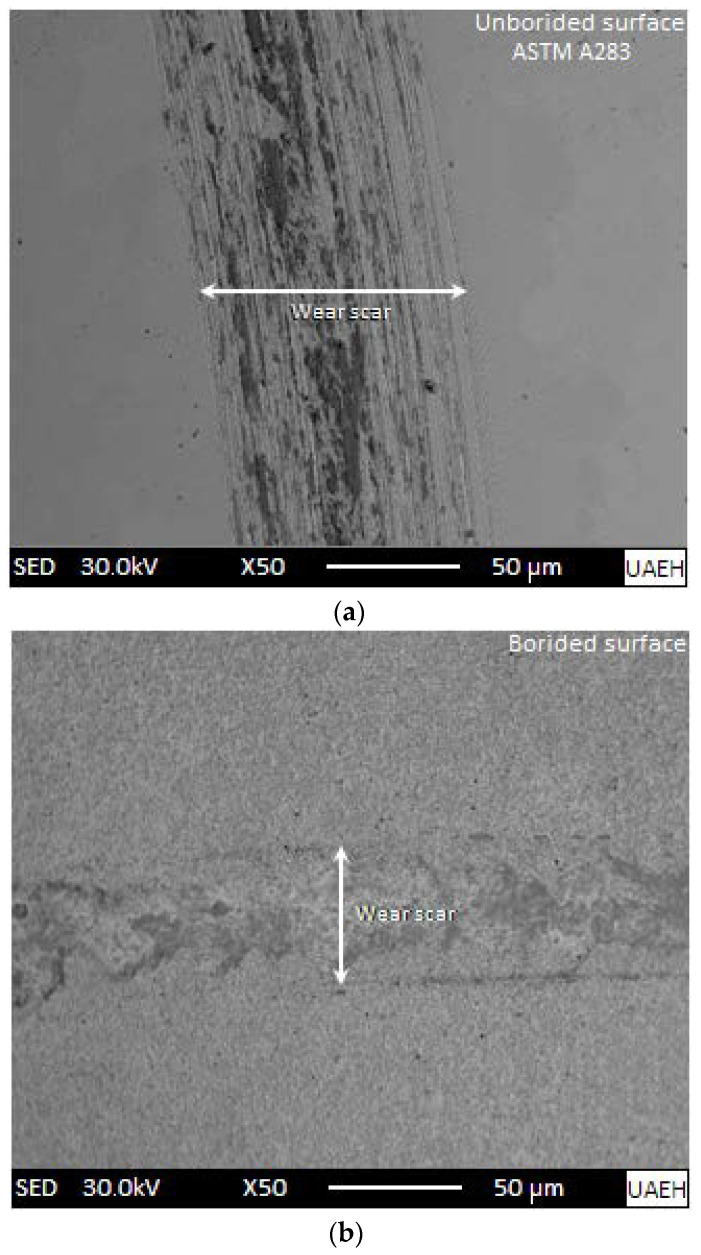
Wear tracks obtained on the surfaces of (**a**) untreated substrate and (**b**) boronized ASTM A283 steel for 8 h at 1273 K.

Figure 12 illustrates the scanning electron microscopy (SEM) images of the generated wear tracks after the tribological tests visible on the surfaces of the untreated substrate and the boronized ASTM A283 steel at 1273 K for 8 h. In Figure 12a, spalling areas are visible with the presence of scratch lines on the surface of the untreated substrate. Furthermore, the presence of wear debris can cause more damage to the plowed-up surface along the wear track under the action of normal load. Figure 12b gives the SEM micrograph of the formed wear track on the borided surface of ASTM A283 steel. The tested surface did not undergo a severe action of wear and some areas of the surface were subjected to an intense plastic deformation with the preservation of its integrity. This is due to the presence of the hard boride layer on its surface limiting its wear degradation. It is important to note that the width of the wear track is smaller for the treated surface compared to the untreated one because of the formation of the Fe_2_B layer on it.

**Figure 13:**
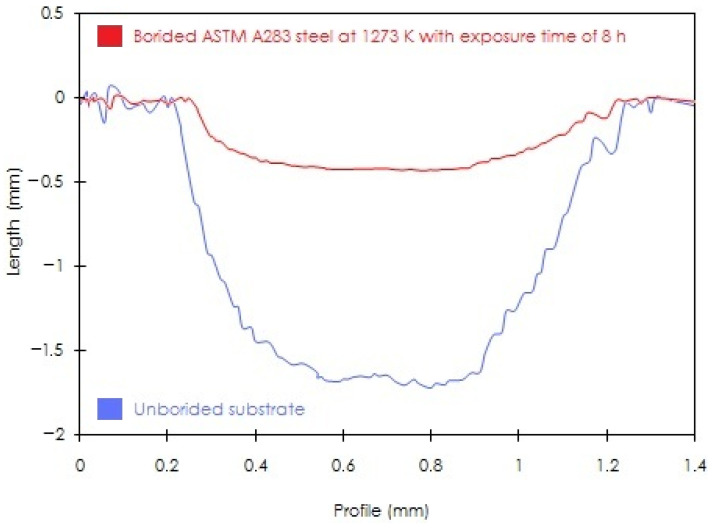
Profiles of wear tracks obtained on the surfaces of untreated substrate and boronized ASTM A283 steel for 8 h at 1273 K.

Figure 13 presents the obtained profiles of in-depth wear tracks generated on the surfaces of substrates and treated ASTM A283 steel at 1273 K during 8 h. The selected boriding parameters resulted in the thickest layer thickness. It is noted that the substrate surface has undergone a severe wear leaving behind a deeper wear track in comparison with that appearing on the treated surface by boriding. Thus, the presence of the hardened surface layer reduced the severity of sliding wear by accommodating plastically the imposed mechanical deformation under the action of applied normal load. The same trend was observed in other studies [6,12,13,36,38].

It is essential to mention that the profilometry test provides a cross-sectional view of the surface’s sliding direction of the topographic profile by contact. Figure 12 provides the SEM images, the wear track of the borided sample is slightly less wide compared to the untreated substrate. However, in Figure 13, the profiles of the wear tracks appear to coincide in width; possibly, what is happening is caused by the particles released during scratching accumulating considerably, which causes an increase in the width of the wear track but does not contribute to the depth.

### 3.6. Assessing the Minimum Boron Energy Value in Diiron Boride for ASTM A283 Steel

**Figure 14:**
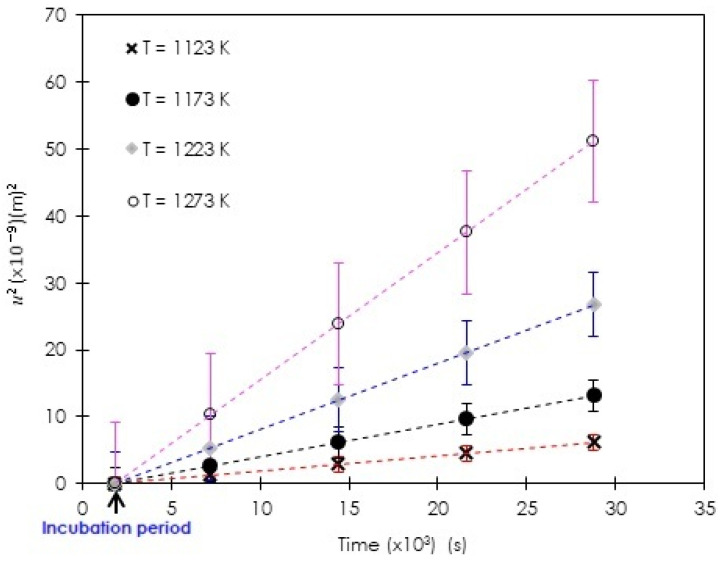
The plot of the square of Fe_2_B layer thickness versus exposure time.

The values of boron diffusion coefficients in Fe_2_B were obtained by employing the steady-state diffusion model through Equation (7) and the integral method through Equation (21). Such a calculation required the determination of the parabolic growth constants listed in Table 1. These values were obtained from the slopes (m=tanθ) of the plot of the square of Fe_2_B layer thickness versus time as represented in Figure 14 based on the present kinetic data on pack-boronized ASTM A283 steel.

It is worth noticing that within the chosen temperature range, the incubation times are pretty similar.

**Figure 15:**
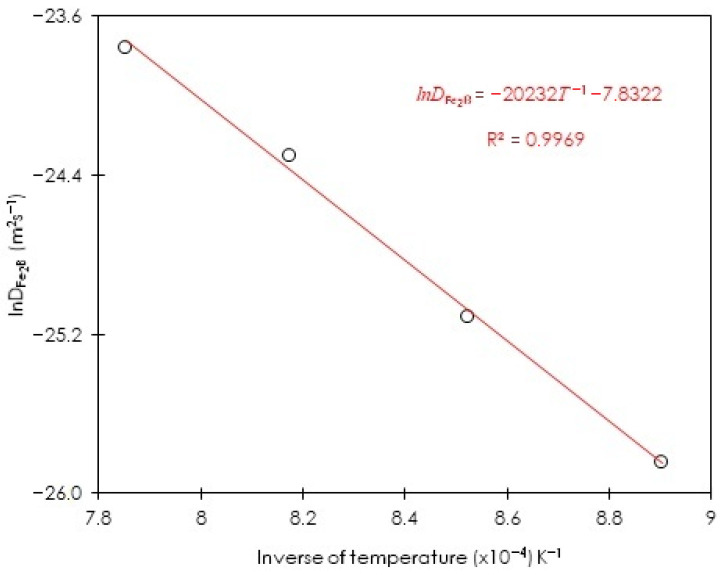
Arrhenius relationship between the estimated boron diffusivity in Fe_2_B and the process temperature by the first approach (steady-state diffusion model).

In Figure 15, the plot of the natural logarithm of estimated boron diffusivities in Fe_2_B is shown using the first approach (steady-state diffusion model) employing Equations (6) and (7) versus the inverse of process temperature.

Equation (22) about the boron diffusion coefficient in Fe_2_B was obtained by a linear regression from the data of Figure 15, as follows:

(22) $$D_{Fe_{2}B}=3.9\times 10^{-\;4}\exp\left(-\frac{168\;kJ\cdot mol^{-1}}{RT}\right).$$

where R(=8.314 J/mol⋅K) is a physical constant and T corresponds to the temperature.

**Figure 16:**
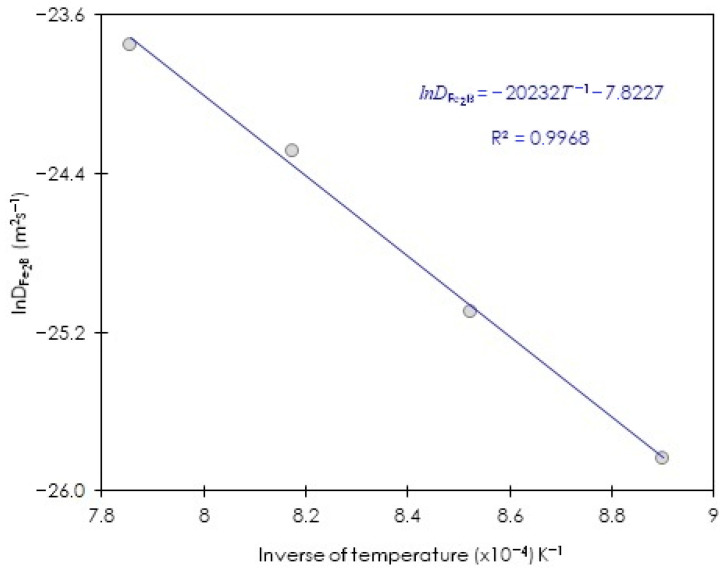
Arrhenius relationship between the estimated boron diffusivity in Fe_2_B and the process temperature by the second approach (the integral diffusion model).

Figure 15 plots $lnD_{Fe_{2}B}$ versus the inverse of the temperature; the obtained slope allows us to associate it with the minimum boron energy value in Fe_2_B for ASTM A283 steel which is equal to 168 kJ∙mol^−1^. The same analysis was performed for the second approach (the integral diffusion model). In Figure 16, the plot of the natural logarithm of estimated boron diffusivities in Fe_2_B is shown by employing Equations (6) and (21) versus the inverse of process temperature.

Equation (23) about the boron diffusion coefficient in Fe_2_B was obtained by a linear regression from the data of Figure 16, as follows:

(23) $$D_{Fe_{2}B}=4.0\times 10^{-\;4}\exp\left(-\frac{168\;kJ\cdot mol^{-1}}{RT}\right).$$

Figure 16 plots $lnD_{Fe_{2}B}$ versus the inverse of the temperature, the obtained slope allows us to associate it with the minimum boron energy value in Fe_2_B for ASTM A283 steel which is equal to 168 kJ∙mol^−1^, exactly the same as the first approach (steady-state diffusion model).

## 4. Discussion

### Analysis of Mathematical Diffusion Models

From the two models proposed (the steady-state and the integral diffusion method, also known as Goodman’s method) to study the growth kinetics of the boride layers formed on the surface of ASTM A283 steel, it was observed that, for both models, the value of the activation energy (the minimum boron energy value) is precisely the same ($Q=168\;kJ\cdot mol^{-1}$). Only slight variation in the pre-exponential factors was found for both models (first model ($D_{0}=3.9\times 10^{-\;4}\;m/s^{2}$) and second model ($D_{0}=4.0\times 10^{-\;4}\;m/s^{2}$)). To find the reason for this coincidence between the two models, Equation (21) was analyzed, which corresponds to the second diffusion model (the integral diffusion model). The $\sqrt{1+4\left(C_{up}^{Fe_{2}B}-C_{low}^{Fe_{2}B}/C_{up}^{Fe_{2}B}+C_{low}^{Fe_{2}B}-2C_{0}\right)}$ term is developed as a Taylor series as follows [36]:

(24) $$\begin{array}{l} \sqrt{1+4\left(\frac{C_{up}^{Fe_{2}B}-C_{low}^{Fe_{2}B}}{C_{up}^{Fe_{2}B}+C_{low}^{Fe_{2}B}-2C_{0}}\right)}\approx 1+2\left(\frac{C_{up}^{Fe_{2}B}-C_{low}^{Fe_{2}B}}{C_{up}^{Fe_{2}B}+C_{low}^{Fe_{2}B}-2C_{0}}\right)\\ -\frac{1}{2}{\left(\frac{C_{up}^{Fe_{2}B}-C_{low}^{Fe_{2}B}}{C_{up}^{Fe_{2}B}+C_{low}^{Fe_{2}B}-2C_{0}}\right)}^{2}+\frac{1}{4}{\left(\frac{C_{up}^{Fe_{2}B}-C_{low}^{Fe_{2}B}}{C_{up}^{Fe_{2}B}+C_{low}^{Fe_{2}B}-2C_{0}}\right)}^{3}-\cdot \cdot \cdot . \end{array}$$

According to the numerical value of the $\sqrt{1+4\left(C_{up}^{Fe_{2}B}-C_{low}^{Fe_{2}B}/C_{up}^{Fe_{2}B}+C_{low}^{Fe_{2}B}-2C_{0}\right)}$, Equation (24) can be rewritten as

(25) $$\sqrt{1+4\left(\frac{C_{up}^{Fe_{2}B}-C_{low}^{Fe_{2}B}}{C_{up}^{Fe_{2}B}+C_{low}^{Fe_{2}B}-2C_{0}}\right)}\approx 1+2\left(\frac{C_{up}^{Fe_{2}B}-C_{low}^{Fe_{2}B}}{C_{up}^{Fe_{2}B}+C_{low}^{Fe_{2}B}-2C_{0}}\right).$$

By substituting Equation (25) into Equation (21), we obtain

(26) $$\varepsilon^{2}=\frac{1+2\left(\frac{C_{up}^{Fe_{2}B}-C_{low}^{Fe_{2}B}}{C_{up}^{Fe_{2}B}+C_{low}^{Fe_{2}B}-2C_{0}}\right)-1}{2}=\frac{C_{up}^{Fe_{2}B}-C_{low}^{Fe_{2}B}}{C_{up}^{Fe_{2}B}+C_{low}^{Fe_{2}B}-2C_{0}}.$$

Equation (26) coincides with Equation (7); this result implies the equivalence between both diffusion models. Table 2 shows different values of boron activation energies (the minimum boron energy value) in some steels [9,13,38,39,40] taken from the literature. The observed differences in the values of activation energies are related to various factors including the chemical compositions of steels, the nature of the boriding process, the selected boriding parameters, the approach used for the calculation, the physical state of the boron source, and the chemical reactions governing the supply of active boron. For example, Gunes et al. [9] employed the plasma-paste boriding for treating the AISI 8620 steel. The estimated values of activation energies were directly dependent on the chemical composition of the boron-paste mixtures from 99 to 108 kJ·mol^−1^. This process required a process temperature below 800 °C and a lower value of activation energy due to a high mobility of radical and neutral species present in the activated plasma. Morgado-González et al. [13] pack-boronized the ASTM A1011 steel by using the same chemical composition of powder mixture expressed in weight percent (i.e., 33.5% B_4_C, 5.4% KBF_4_, and 61.1% SiC) and obtained by the MDC method, a value of 159 kJ·mol^−1^ close to that deduced from the present work with the integral method.

Kayali and Kara [37] employed the pack-boriding method for surface hardening of hardox-450 steel with Ekabor-2 to form diiron boride layers between 1123 and 1273 K. They found an activation energy of 158 kJ·mol^−1^ which is very comparable to our estimated value. Kartal et al. [41] performed the electrochemical boriding on AISI 1018 steel in the electrolyte containing 90 wt.% borax and 10 wt.% sodium carbonate at a current density of 200 mA·cm^−2^ for an even shorter time of 5 min up to 2 h. They found an activation energy of 173 ± 8 kJ·mol^−1^. Sen et al. [42] treated the AISI 5140 steel by employing a slurry salt bath constituted of borax and ferro-silicon to produce iron boride layers. The value determined for the minimum boron energy value (=223 kJ mol^−1^) deduced from the parabolic law of growth was deemed higher compared to the other values [9,13,37,41,43,44] displayed in Table 2. Recently, Arslan et al. [43] developed a novel technique of boriding called pulse current integrated CRTD-Bor in a molten salt electrolysis. Via this technique, it is feasible to adjust the duty cycle for a fixed current density to produce the Fe_2_B layer instead of a dual phase boride layer (FeB + Fe_2_B). The advantage of this boriding method over all the other processes [9,13,38,40,41,42,44,45] is the shortening of time duration to a few minutes. Therefore, the diminution of energy has a positive impact on the reduction of activation energy of this process (=39 kJ mol^−1^). Such a value is the lowest one compared to those of Table 2.

Milinović et al. [44] pack-borided the C45 steel by using Durborid 3 as a boronizing agent in the range of 1143 to 1243 K and found a value of activation energy equal to 199 kJ·mol^−1^ higher to that found in this work and resulting from the difference in the chemical composition of the boriding agent. Su et al. [45] investigated adding 5 wt.% of Nd_2_O_3_ to the powder mixture during the pack-boriding of AISI 1045 steel. They found that the presence of Nd_2_O_3_ oxide as a chemical catalyst accelerates the diffusivity rate of boron atoms (B) by reducing the required minimum boron energy value for the process by approximately 70%. Fang et al. [40] used the pack-boriding for a synthetic powder metallurgy (PM) iron alloy containing 3 wt.% of carbon. The boriding agent used was composed of 10 wt.% B_4_C, 80 wt.% SiC, 5 wt.% KBF_4_, and 5 wt.% C to form the bilayer (FeB + Fe_2_B) with the occurrence of some porosity. The calculated activation energy in this case was equal to 38.8 kJ mol^−1^.

**Figure 17:**
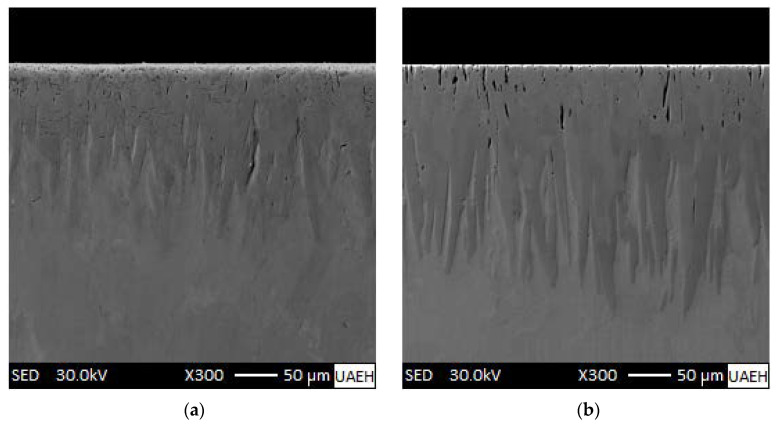
SEM images of the cross-sectional views of boride layers corresponding to two boriding temperatures for 9 h of treatment: (**a**) 1223 K and (**b**) 1273 K.

In Figure 17, the SEM images of cross-sectional views of treated samples by boriding according to two boriding conditions (at 1223 and 1273 K for a time duration of 9 h) are displayed. Table 3 contains the layers’ thicknesses determined experimentally at 1223 and 1273 K for 9 h and the calculated ones with Equation (6) for a $C_{up}^{Fe_{2}B}$ in Fe_2_B equal to 9 wt.% with an incubation period of 1796 s for Fe_2_B. The estimated Fe_2_B layers’ thicknesses agreed with the experimental data when making a comparison.

.

Additionally, the two diffusion models proposed for the estimation of the Fe_2_B layer thickness cannot be extended to the case of carburizing or nitriding, although carbon and nitrogen are interstitial elements that can easily diffuse into the substrate since the concentration of carbon or nitrogen on the substrate surface for both thermochemical treatments varies for the treatment temperature; this implies that the diffusion coefficient is a function of the concentration [46,47]. However, the modeling of the growth of Fe_2_B boride layers is a relevant tool to optimize the parameters involved in the process to achieve the desired thickness ($u(t)=1.9\times 10^{-2}D_{Fe_{2}B}^{1/2}{\left(t-t_{0}^{Fe_{2}B}\right)}^{1/2}$) of the boride layer according to the practical use of the treated parts. Recently, several works related to the integral method have been applied to study the growth kinetics of Fe_2_B layers on ARMCO iron, AISI 12L14, AISI P20, AISI 4150, and AISI S1 [48,49,50,51,52]. In this diffusion model, an analytical solution was obtained from a system formed by differential algebraic equations (DAE) to obtain an expression to determine the diffusion coefficient of boron in the Fe_2_B phase. However, for some readers unfamiliar with differential algebraic equations, the integral model will be challenging. In this sense, the comparison of the two diffusion models that we proposed (method without time dependence and integral method) allows us to establish an equivalence between both models, thus confirming that the model without time dependence is a practical and straightforward tool to study the growth kinetics of boride layers. It is also important to note that the model without time dependence can be extended to determine the diffusion coefficients of a double layer (FeB + Fe_2_B).

## 5. Conclusions

The main objective of this study was to compare two mathematical models of mass transfer (the steady-state diffusion model and the integral diffusion model) from the kinetic growth of Fe_2_B layers formed on the surface of the selected substrate. In the first model, the mass balance equation ($(C_{up}^{Fe_{2}B}+C_{low}^{Fe_{2}B}-2C_{0}/2){\left(dx/dt\right)|}_{x\;=\;u}=-D_{Fe_{2}B}{\left(dC_{Fe_{2}B}(x)/dx\right)|}_{x\;=\;u}$), the concentration profile without time dependence ($C_{Fe_{2}B}(x)=((C_{low}^{Fe_{2}B}-C_{up}^{Fe_{2}B})x/u)+C_{up}^{Fe_{2}B}$), and parabolic growth law ($u(t)=2\varepsilon D_{Fe_{2}B}^{1/2}{\left(t-t_{0}^{Fe_{2}B}\right)}^{1/2}$) along the boride layers (Fe_2_B) were considered to determine the $\varepsilon \;(=9.5\times 10^{-\;3})$ constant. In the second model, the second Fick’s law ($D_{Fe_{2}B}\partial^{2}C_{Fe_{2}B}(x,t)/\partial x^{2}=\partial C_{Fe_{2}B}(x,t)/\partial t$), a preliminarily defined parabolic profile with time dependence ($C_{Fe_{2}B}(x,t)=b(t){\left(u-x\right)}^{2}+a(t)(u-x)+c$), and the mass balance equation with time dependence ($(C_{up}^{Fe_{2}B}+C_{low}^{Fe_{2}B}-2C_{0}/2){\left(dx/dt\right)|}_{x\;=\;u}=-D_{Fe_{2}B}{\left(\partial C_{Fe_{2}B}(x,t)/\partial x\right)|}_{x\;=\;u}$) were used to determine the $\varepsilon \;(=9.4\times 10^{-\;3})$ constant. The results allow us to conclude that there is equivalence between the two models. It seems that the second mathematical model (the integral diffusion model) is more reliable and sophisticated because it considers the influence of time. The minimum boron energy value was calculated as 168 kJ∙mol^−1^ for Fe_2_B in ASTM A283 steel by using both approaches. Lastly, the experimental layers’ thicknesses were concordant with the theoretical values. In addition, the substrate was subjected to pack-boriding using a boron yielding agent containing boron carbide (B_4_C), potassium tetrafluoroborate (KBF_4_), and silicon carbide (SiC) in the range of 1123–1273 K for 2–8 h. The jagged growth was a characteristic of formed layers with thicknesses ranging from 35 ± 8 to 227 ± 35 µm. The boride layers had a sufficient cohesive quality with respect to the substrate complying with HF2 and HF4 categories. However, the best interfacial cohesion resulting from the Daimler-Benz Rockwell C tests corresponded to the boriding condition of 1123 K for 2 h. This result is due to the formation of a thinner layer compared to the one formed with a temperature of 1273 K and 8 h of exposure time. The borided sample was wear resistant compared to the untreated sample owing to the presence of a Fe_2_B layer with a maximum friction coefficient of 0.29 versus 0.6 for the untreated surface. The wear mechanism occurring at the surface of the borided sample is dominated by plastic deformation, whereas it is of adhesive nature for the untreated surface (with the presence of scratching and a clear spalling).

## Figures and Tables

**Table 1:** Empirically obtained $4\varepsilon^{2}D_{Fe_{2}B}$ values with the incubation periods based on our kinetic data.

Boriding Temperature T (K)	$\mathrm{Experimental}\;\mathrm{Kinetic}\;\mathrm{Constant}\;4\varepsilon^{2}D_{Fe_{2}B}$	Incubation Period (s)
1123	2.3 × 10^−13^	1798
1173	4.7 × 10^−13^	1796
1223	1.1 × 10^−12^	1796
1273	1.8 × 10^−12^	1795

**Table 2:** Obtained boron activation energies in case of some treated steels.

Type of Steel	Boriding Process	Activation Energy (kJmol^−1^)	Temperature Range of Investigation (K)	Approach for Calculation	References
AISI 8620	Plasma-paste boriding	(FeB + Fe_2_B) 99–108	973–1073	Parabolic growth law	[9]
ASTM A1011	Powder	(Fe_2_B) 159	1123–1273	Mean diffusion coefficient method	[13]
Hardox–450	Powder	(Fe_2_B) 158	1123–1223	Parabolic relation	[37]
PM Iron alloy at 3 wt.%C	Powder	(FeB + Fe_2_B) 164	1123–1223	Parabolic relation	[40]
AISI 1018	Electrochemical boriding	(FeB + Fe_2_B) 173 ± 8	1123–1273	Parabolic relation	[41]
AISI 5140	Salt bath	(FeB + Fe_2_B) 223	1123–1273	Parabolic relation	[42]
Low carbon steel	Pulse current integrated CRTD-Bor	Dominant Fe_2_B 38	1123–1323	Parabolic relation	[43]
C45 steel	Powder	(FeB + Fe_2_B) 199	1143–1243	Parabolic relation	[44]
AISI 1045	Powder	(FeB + Fe_2_B) 198–137 with wt.% Nd_2_O_3_	1053–1213	Parabolic relation	[45]
ASTM A283	Powder	(Fe_2_B) 168	1123–1273	Steady-state diffusion model and Integral method	This work

**Table 3:** Experimental data for layer thicknesses produced at 1223 and 1273 K during 9 h compared with the calculated values from Equation (6) for a $C_{up}^{Fe_{2}B}=9\;wt.\%$.

Boronizing Conditions T (K)	Experimental Values of Fe_2_B Layers’ Thicknesses (μm)	Calculated Fe_2_B Layers’ Thicknesses (μm)
1223 K for 9 h	181 ± 27	175
1273 K for 9 h	241 ± 38	246

## Data Availability

The authors confirm that the data supporting the findings of this study are available within the article.
